# A randomised, double-blind, placebo-controlled study to evaluate the safety and efficacy of lamotrigine in the maintenance treatment of Chinese adult patients with bipolar I disorder

**DOI:** 10.1186/s40345-022-00266-4

**Published:** 2022-08-01

**Authors:** Ling Zhang, Honggeng Zhang, Lu-xian Lv, Qingrong Tan, Xiufeng Xu, Jian Hu, Lu Zi, James Cooper, Abhay Phansalkar, Gang Wang

**Affiliations:** 1 The National Clinical Research Center for Mental Disorders & Beijing Key Laboratory of Mental Disorders, Beijing Anding Hospital , Capital Medical University, 100088 Beijing, China; 2 Advanced Innovation Center for Human Brain Protection , Capital Medical University , 100069 Beijing, China; 3 Department of Psychiatry, Brains Hospital of Hunan Province, Changsha, China; 4 Department of Psychiatry, Henan Mental Hospital, The Second Affiliated Hospital of Xinxiang Medical University, Xinxiang, People’s Republic of China; 5 Department of Psychiatry, Xijing Hospital, The Fourth Military Medical University, Xi’an, China; 6 Department of Psychiatry, First Affiliated Hospital of Kunming Medical University, Kunming, People’s Republic of China; 7 The First Affiliated Hospital of Harbin Medical University, Nangang, Harbin, People’s Republic of China; 8 GlaxoSmithKline R&D Co., Ltd, Shanghai, China; 9 GlaxoSmithKline R&D Ltd, Brentford, Middlesex UK; 10 GlaxoSmithKline India Global Services Private Ltd, Mumbai, India

**Keywords:** Maintenance, Mood episode, Prevention, Recurrence, Relapse, Bipolar I disorder, Lamotrigine

## Abstract

**Background:**

Lamotrigine is approved as a maintenance therapy for bipolar I disorder in many countries, including China in 2021. This study evaluated the efficacy and safety of lamotrigine in controlling relapse and/or recurrence of mood episodes in Chinese patients with bipolar I disorder.

**Methods:**

Patients aged ≥ 18 years with bipolar I disorder who met response criteria (Clinical Global Impression–Severity [CGI-S] score of ≤ 3 for ≥ 4 consecutive weeks) during treatment with lamotrigine in a 6–16 week open-label (OL) phase, and who were maintained for ≥ 1 week on lamotrigine 200 mg/day monotherapy, were randomised (1:1) to continue receiving lamotrigine 200 mg/day or switch to placebo in a 36-week randomised double-blind (RD) phase. The primary efficacy outcome measure was time from entry into the RD phase to intervention for relapse and/or recurrence of a mood episode (TIME). Post hoc analyses assessed the impact of OL baseline mood severity on TIME. Safety assessments were conducted throughout the study.

**Results:**

Of 420 patients treated in the OL phase, 264 were randomised to receive lamotrigine (n = 131) or placebo (n = 133). Overall, 112 patients had an intervention for relapse and/or recurrence of a mood episode (lamotrigine, n = 50/130 [38.5%]; placebo, n = 62/133 [46.6%]), with no significant difference in TIME between groups (adjusted hazard ratio [95% confidence interval (CI)] 0.93 [0.64, 1.35]; p = 0.701). Post hoc analyses indicated a significant difference in TIME, favouring lamotrigine over placebo, for patients with baseline CGI-S score ≥ 4 (hazard ratio [95% CI] 0.52 [0.30, 0.89]; p = 0.018) and with baseline Hamilton Depression Rating Scale ≥ 18 or Young Mania Rating Scale ≥ 10 (0.44 [hazard ratio [95% CI] 0.25, 0.78]; p = 0.005). Lamotrigine was well tolerated with no new safety signals.

**Conclusions:**

Lamotrigine was not significantly superior to placebo in preventing relapse and/or recurrence of mood episodes in this study of Chinese patients with bipolar I disorder but post hoc analyses suggested a therapeutic benefit in patients with moderate/severe mood symptoms at baseline. The discrepancy between these findings and the positive findings of the pivotal studies may be attributable to the symptom severity of the bipolar patients recruited, a high dropout rate, and the comparatively short duration of the RD phase rather than race/ethnicity differences.

*Clinical trial registration* ClinicalTrial.gov Identifier NCT01602510; 21st May 2012; https://clinicaltrials.gov/ct2/show/NCT01602510.

**Supplementary Information:**

The online version contains supplementary material available at 10.1186/s40345-022-00266-4.

## Introduction

Bipolar disorder is a severe and chronic psychiatric disorder, characterised by both depressive and manic/hypomanic episodes with a highly recurrent course (Grande et al. 2016). As of 2017, bipolar disorder was estimated to affect 46 million people globally (GBD 2017). A recent meta-analysis of bipolar disorder I prevalence in China reported a lifetime prevalence of 0.09%, which is comparatively lower than observed in international populations (Zhang et al. 2017). On average, patients with bipolar I disorder experience two mood episodes per year (Tondo et al. 2017), and maintenance treatment aimed at preventing future episodes is critical to their clinical management (Grande et al. 2016).

Lamotrigine, an inhibitor of voltage-sensitive sodium channels, is approved in the United Stated (US) (target dose 200 mg/day) for the maintenance treatment of bipolar I disorder to delay the time to occurrence of mood episodes in patients treated with standard therapy for acute mood episodes (FDA 2020). It is also approved for the prevention of bipolar depression in more than 30 countries worldwide (Prabhavalkar et al. 2015; Electronic Medicines Compendium 2020). Major international and Chinese guidelines for the management of bipolar disorder recommend lamotrigine as a maintenance treatment to prevent relapse/recurrence of mood episodes (Goodwin et al. 2016; Yatham et al. 2018; Wang et al. 2018; Grunze et al. 2013).

Pivotal studies for the international approval of lamotrigine treatment for bipolar I disorder demonstrated prevention of mood episodes for recently depressed patients (Calabrese et al. 2003) and for recently manic/hypomanic patients (Bowden et al. 2003) with bipolar I disorder. Several systematic reviews and meta-analyses have confirmed the beneficial effects of lamotrigine as maintenance therapy for prevention of depressive episodes in patients with bipolar disorder (Oya et al. 2019; Kishi et al. 2021; Miura et al. 2014). The efficacy of lamotrigine was found to be comparable with lithium (Kishi et al. 2021; Licht et al. 2013). Lamotrigine has also proven beneficial in controlling the relapse or recurrence of bipolar episodes in Chinese populations (Chen 2010; Shi et al. 2008).

Until recently, fewer treatment options were available in China for the prevention of depression than for the treatment of mania, and mania treatments with a better safety profile than lithium, valproate and anti-psychotics were needed. Lamotrigine was approved in 2021 for use in China as a maintenance treatment for the control of relapse and/or recurrence of mood episodes, before which its use was limited to the treatment of patients with epilepsy. The present placebo-controlled study evaluated the efficacy and safety of lamotrigine 200 mg/day for preventing relapse and/or recurrence of a manic, hypomanic, mixed or depressive episode in Chinese adult patients with bipolar I disorder.

## Methods

### Study design

This was a randomised, double-blind, placebo-controlled, parallel-group, multi-centre, fixed-dose study in adult patients (aged ≥ 18 years) with bipolar I disorder conducted from August 2012 to December 2015 at 21 centres in China. The study consisted of four phases: screening phase, open-label (OL) phase, randomised double-blind (RD) phase and a follow-up visit (Additional file 1: Fig. S1). Patients meeting the eligibility criteria entered the OL phase to receive lamotrigine monotherapy or adjunctive therapy escalated to a target dose of lamotrigine 200 mg/day monotherapy for up to 16 weeks.

Beginning at Week 7 of the OL phase, patients who met the response criteria (Clinical Global Impressions of Severity [CGI-S] score ≤ 3 maintained for ≥ 4 continuous weeks) and who were maintained for ≥ 1 week on lamotrigine 200 mg/day monotherapy, while demonstrating compliance with the study treatment, were enrolled in the RD phase and randomised (1:1) to lamotrigine 200 mg/day or placebo. Patients who completed 36 weeks of randomised treatment, reached the study endpoint (intervention for relapse and/or recurrence of a mood episode) or withdrew from the study early (including the OL phase and RD phase) were followed up for 14 days after the last dose.

Patients were randomised (1:1) sequentially (block of four) through GlaxoSmithKline’s interactive voice response system, following a computer-generated randomisation schedule. Both investigators and patients were blinded to treatment allocation.

The study was conducted in accordance with the International Conference on Harmonization Good Clinical Practices, applicable country-specific requirements and the ethical principles outlined in the Declaration of Helsinki (2008). The study protocol was approved by an Independent Ethics Committee or Institutional Review Board at each study centre. Written informed consent was obtained from each participant prior to any study-specific procedures.

### Treatment

When lamotrigine was used as adjunctive therapy with valproate during the OL phase, the prespecified starting dose of lamotrigine and subsequent increases in dose were reduced to half; however, when used with carbamazepine, the starting dose of lamotrigine and subsequent dose adjustment rates were doubled. Concomitant antiepileptic drugs and psychotropic medications (except fluoxetine) were permitted for the treatment of mood episodes during the OL phase but were discontinued at least 2 weeks (at least 3 weeks for lithium) prior to entering the RD phase. During the OL and RD phases, the short-term (two to three times/week, duration < 2 weeks) use of chloral hydrate, lorazepam, clonazepam, estazolam or oxazepam was permitted as required for control of agitation, irritability, insomnia and hostile behaviour. No other psychotropic, antidepressant or antimanic drug was permitted unless the patient had reached the study endpoint, at which point study treatment was stopped and one or more of the following psychotropic medications were prescribed: antidepressants, antipsychotics (with or without anticholinergic medications), benzodiazepines (exceeding the above dosages) and anticonvulsants/mood stabilisers. Concomitant medications for co-morbid conditions (including diabetes and hypertension) were permitted at the discretion of the investigator. During the RD phase, lamotrigine (200 mg) or matching placebo tablets were administered orally, once daily in the evening.

### Participants

Patients of either gender (aged ≥ 18 years) were recruited from inpatient or outpatient clinics. Patients entering the OL phase were diagnosed (using medical records and clinical interviews) with bipolar I disorder and had either a current or most recent depressed (296.5×), hypomanic (296.40), manic (296.4×), or mixed (296.6×) episode (as defined by Diagnostic and Statistical Manual of Mental Disorders-IV [DSM-IV] criteria) within the last 60 days. Patients diagnosed with 296.5 × must have had at least one well-documented manic, hypomanic or mixed episode, as defined by DSM-IV criteria, within 3 years of enrolment. Patients diagnosed with 296.40, 296.4 × or 296.6 × must have had at least one well-documented additional manic, hypomanic or mixed episode and one depressed episode, as defined by DSM-IV criteria, within 3 years of enrolment. Patients were excluded from the OL phase if they met DSM-IV criteria for rapid cycling in the 12-month period prior to enrolment; had significant DSM-IV Axis II diagnosis; had current or previous diagnosis of an Axis I disorder with the exception of bipolar disorder, had signs or symptoms of psychosis, were at suicidal risk; had a history of substance abuse or dependence; had received fluoxetine within 4 weeks prior to the OL phase, used oral contraceptives or other hormonal preparations containing oestrogen within 2 weeks prior to OL phase entry; or had a history or current diagnosis of epilepsy, were morbidly obese (body mass index > 35 kg/m^2^) or were pregnant or lactating women. Factors leading to withdrawal before entry to the RD phase included signs or symptoms of psychosis, the need for treatment of a manic or mixed episode during the OL phase (with new courses of lithium/psychotropic drugs or other drugs with a half-life greater than 14 days), becoming actively suicidal and/or having a score ≥ 3 on item 3 of the Hamilton Depression Rating Scale (HAMD), or testing positive for an illicit drug on laboratory analysis administered before randomisation or alcohol abuse/addiction.

Patients were withdrawn from the study if they required intervention for a relapse and/or recurrence of a mood episode (primary endpoint during the RD phase); had a medically relevant adverse event (AE) or intercurrent illness or were pregnant; demonstrated significant non-compliance with the protocol or investigational treatment; had an inability to tolerate the drug; developed a rash or hypersensitivity reaction; had a prolonged QT interval (QTc > 500 ms or uncorrected QT > 600 ms or, in cases with bundle branch block, QTc > 530 ms based on average QTc value of triplicate electrocardiograms [ECGs]); or discontinued treatment.

### Assessments and outcome measures

Demographic and baseline characteristics were assessed by study investigators at screening or baseline. During the RD phase, patients were assessed at weekly intervals for the first month, biweekly intervals for the second month and then at monthly intervals for up to 36 weeks. The HAMD, Young Mania Rating Scale (YMRS), Clinical Global Impressions of Improvement (CGI-I), CGI-S and Global Assessment Scale (GAS) were used as indices for intensity and duration of mood symptoms.

The primary endpoint was intervention for relapse and/or recurrence of a mood episode, defined as first prescription of any additional pharmacotherapy or electroconvulsive therapy necessary for the treatment of a relapse and/or recurrence of a depressive, manic, hypomanic or mixed episode. The primary efficacy outcome measure was the time from entry into the RD phase to intervention for relapse and/or recurrence of a mood episode (TIME). Secondary efficacy outcome measures included: time to intervention for manic, hypomanic or mixed episode (TIMan); time to intervention for depressive episode (TIDep); overall survival in the study (TIME-SIS); changes from baseline to Week 36 in CGI-S (Guy 1976), CGI-I (Guy 1976), HAMD (Hamilton 1960), YMRS (Young et al. 1978), GAS (Endicott et al. 1976); and change in weight from baseline during the RD phase. Safety assessments included: monitoring of AEs, including treatment-emergent adverse events (TEAEs); clinical laboratory tests; vital signs; ECGs; physical examinations; and suicidality, as determined by the Columbia Suicide Severity Rating Scale (C-SSRS). Patient-reported quality of life changes were assessed at each visit using the Short Form 36 (SF-36) questionnaire (Garratt et al. 1993). The eight domains of the SF-36 were physical functioning, role physical, role emotional, bodily pain, vitality, mental health, social functioning and general health. The SF-36 was administered by well-trained personnel who passed the consistency evaluation with a certification record.

### Statistical analysis

Based on previous studies (Calabrese et al. 2003; Bowden et al. 2003), the median time to intervention was 97 days in the lamotrigine group and 58 days in the control group. To detect a treatment difference of the same magnitude in the current study, a target sample size of 178 patients (89 patients in each group) was estimated to provide 90% power at the significance level of 0.05. The minimum regulatory requirement specified by the State Food and Drug Administration of China was 100 pairs of patients. Assuming a 20% dropout rate, at least 250 patients were required for the RD phase (125 patients in each group). Considering 60% of patients who entered the OL phase would be randomised to RD phase, the total number of patients to be included in the OL phase was determined to be 416.

The full analysis population (FAP) for the OL and RD phases included all patients who received at least one dose of the study medication and provided at least one post-baseline efficacy/health outcome assessment during the OL and RD phases, respectively. The safety population included all patients who received at least one dose of the study medication in the corresponding phase (OL or RD phase). No interim analyses were planned or performed for this study.

All analyses were conducted using SAS software (Version 9, SAS Inc., Cary, NC, USA). A superiority test was performed to compare efficacy between two groups. The primary efficacy outcome of TIME was assessed using the Cox proportional hazards regression model, including site and CGI-S baseline score as covariates. Data for patients who discontinued from the study were censored. Kaplan–Meier plots of TIME are presented by treatment group during the RD phase, together with hazard ratios (HR), 95% (two-sided) confidence intervals (CI) and p values. The secondary outcome measures TIME-SIS, TIDep and TIMan were analysed in a similar way, with data censored both for patients who discontinued and for patients who received intervention for a mood episode of the opposite polarity to the relevant endpoint; p values were also provided based on the log-rank tests to describe any significant distribution difference between treatment groups for these endpoints. The between-group comparisons for HAMD, YMRS, CGI-S, CGI-I, GAS scales and body weight (secondary outcome measures) as well as for quality of life scores (SF-36) were performed using analysis of covariance (ANCOVA) or rank sum test.

Post hoc analyses (Cox proportional hazards regression model) were conducted on the FAP to compare the efficacy (TIME) of lamotrigine and placebo in a subgroup of patients with moderate/severe mood symptoms at baseline. One post hoc analysis defined symptoms by CGI-S score (CGI ≥ 4; moderate to severe symptoms), the second defined symptoms by HAMD (≥ 18)/YMRS (≥ 10) scores. A further analysis explored the effect of type of index (most recent) episode (depressive or manic/hypomanic) and first recorded episode (depressive or mixed/manic) on TIME.

Baseline characteristics, including past psychiatric conditions and OL phase lamotrigine exposures were summarised descriptively for each RD treatment population.

## Results

**Fig. 1:**
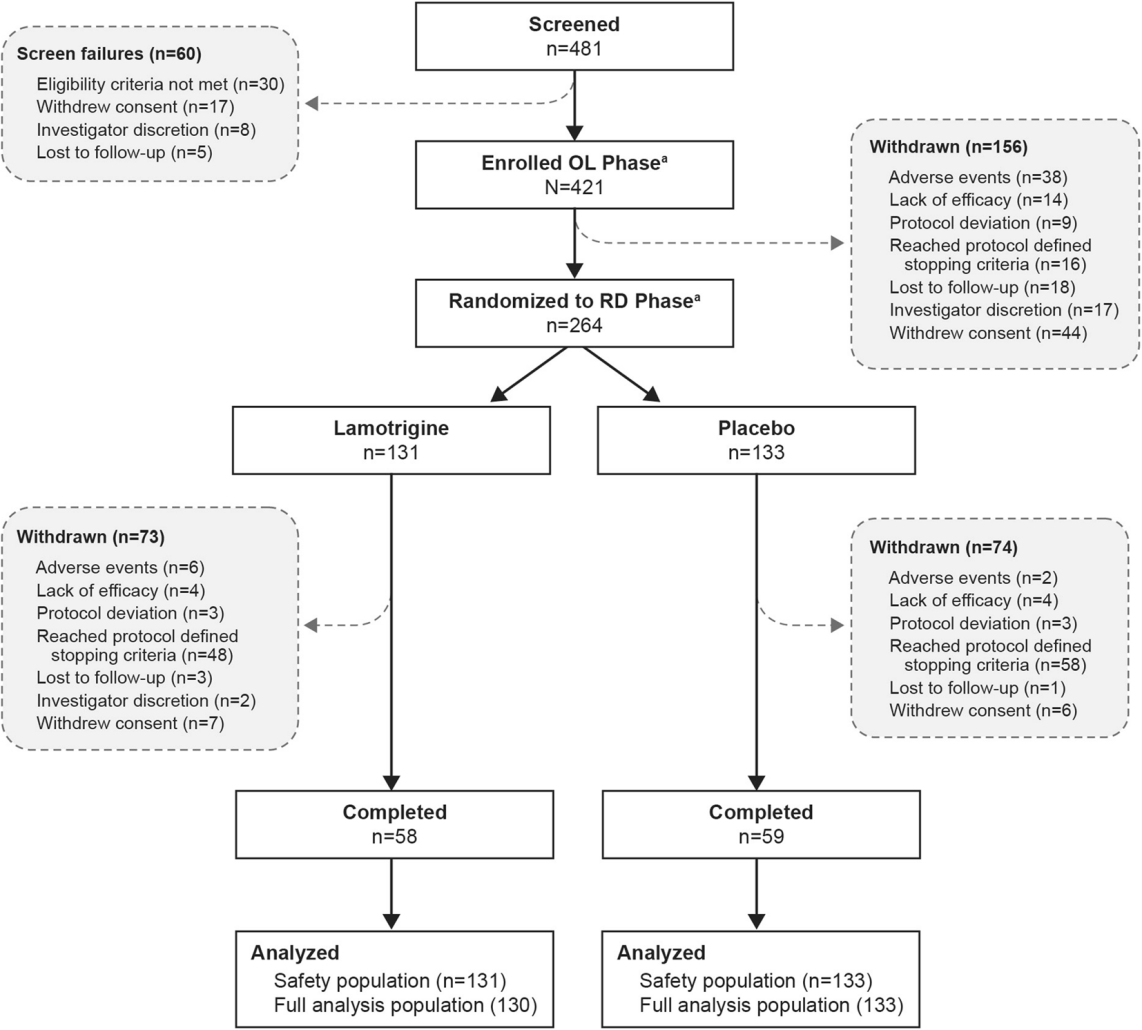
CONSORT flow diagram. **a **One patient was enrolled to OL phase but did not receive any treatment. The stopping criteria was defined as a CGI-S score of ≤ 3 for at least 6 consecutive weeks. CGI-S, Clinical Global Impression–Severity; RD, double-blind; OL, open-label

A total of 421 patients were enrolled into the OL phase and 420 patients received at least one dose of the study medication. Of these 420 patients, 264 were randomised into the RD phase, 263 of whom were included in the FAP (133 in the lamotrigine arm and 130 in the placebo arm); 117 patients (44.3%) completed the study (Fig. 1). A total of 147 (55.7%) patients were withdrawn during the RD phase, most commonly because they had reached protocol-defined withdrawal criteria (n = 106; 40.2%), and withdrawal because an AE was reported for eight patients (3.0%). The demographic and baseline characteristics of both treatment groups in the RD phase were similar (Table 1). The most commonly used concomitant psychiatric medications during the OL phase were lithium carbonate (28.6%) and valproate sodium (28.3%), and during the RD phase were lamotrigine (11.0%) and valproate sodium (8.7%).

**Table 1:** Demographics and baseline characteristics

Characteristic	Open-label phase	Double-blind phase		
	Lamotrigine N = 420	Lamotrigine N = 131	Placebo N = 133	Total N = 264
Age (years)	35.7 (11.7)	34.8 (10.9)	37.4 (12.5)	36.1 (11.8)
Gender, n (%)				
Women	220 (52.4)	75 (57.3)	62 (46.6)	137 (51.9)
Men	200 (47.6)	56 (42.7)	71 (53.4)	127 (48.1)
BMI (kg/m^2^)	24.2 (3.8)	24.0 (3.9)	24.3 (3.8)	24.2 (3.9)
Weight (kg)	67.1 (12.8)	66.9 (13.8)	67.5 (11.9)	67.2 (12.8)
Duration of current episode (weeks)	7.6 (8.0)	7.8 (7.8)	7.7 (7.2)	7.8 (7.5)
Age at onset of first episode (years)				
Depression	29.5 (10.8)^a^	29.5 (9.7)^b^	30.7 (11.6)	30.1 (10.7)^c^
Mixed/manic	30.4 (11.1)^d^	30.4 (10.2)^e^	31.3 (12.1)^f^	30.9 (11.1)^c^
Number of episodes in past 3 years				
Mania	1.1 (0.73)^g^	1.2 (0.68)	1.1 (0.70)^f^	1.1 (0.69)^h^
Hypomania	0.1 (0.32)^g^	0.1 (0.24)	0.1 (0.44)^f^	0.1 (0.35)^h^
Depression	1.2 (0.76)^g^	1.3 (0.81)	1.2 (0.76)^f^	1.2 (0.79)^h^
Index episode, n (%)				
Mania	152 (36)	43 (33)	52 (39)	95 (36)
Hypomania	10 (2)	3 (2)	2 (2)	5 (2)
Depression	237 (56)	81 (62)	72 (54)	153 (58)
Mixed	21 (5)	4 (3)	7 (5)	11 (4)
HAMD score	11.1 (8.2)^d^	2.7 (3.0)^e^	2.3 (2.5)	2.5 (2.8)
YMRS score	6.7 (9.5)^d^	1.0 (2.0)^e^	0.8 (1.6)	0.9 (1.8)
Suicide attempts, n (%)	34 (8.1)	7 (5.3)	7 (5.3)	14 (5.3)
Concomitant psychiatric medications^9^, n (%)				
Lithium carbonate	120 (28.6)	8 (6.1)	7 (5.3)	15 (5.7)
Valproate sodium	119 (28.3)	12 (9.2)	11 (8.3)	23 (8.7)
Olanzapine	78 (18.6)	8 (6.1)	9 (6.8)	17 (6.4)
Quetiapine fumarate	71 (16.9)	7 (5.3)	5 (3.8)	12 (4.5)
Quetiapine	69 (16.4)	8 (6.1)	7 (5.3)	15 (5.7)
Valproate magnesium	58 (13.8)	5 (3.8)	3 (2.3)	8 (3.0)
Clonazepam	48 (11.4)	1 (0.8)	6 (4.5)	7 (2.7)
Citalopram	31 (7.4)	3 (2.3)	5 (3.8)	8 (3.0)
Alprazolam	23 (5.5)	2 (1.5)	2 (1.5)	4 (1.5)
Aripiprazole	22 (5.2)	1 (0.8)	3 (2.3)	4 (1.5)
Paroxetine	22 (5.2)	1 (0.8)	0	1 (0.4)
Lamotrigine	4 (1.0)	13 (9.9)	16 (12.0)	29 (11.0)

Data are presented as mean (SD), unless otherwise specified

BMI, body mass index; HAMD*,* Hamilton Depression Scale; SD*,* standard deviation; YMRS*,* Young Mania Rating Scale

Analysis population: safety population

^a^n = 415; ^b^n = 129; ^c^n = 262; ^d^n = 416; ^e^n = 130; ^f^n = 132; ^g^n = 419; ^h^n = 263; ^i^More than 5% in any group

### Primary efficacy endpoint

**Fig. 2:**
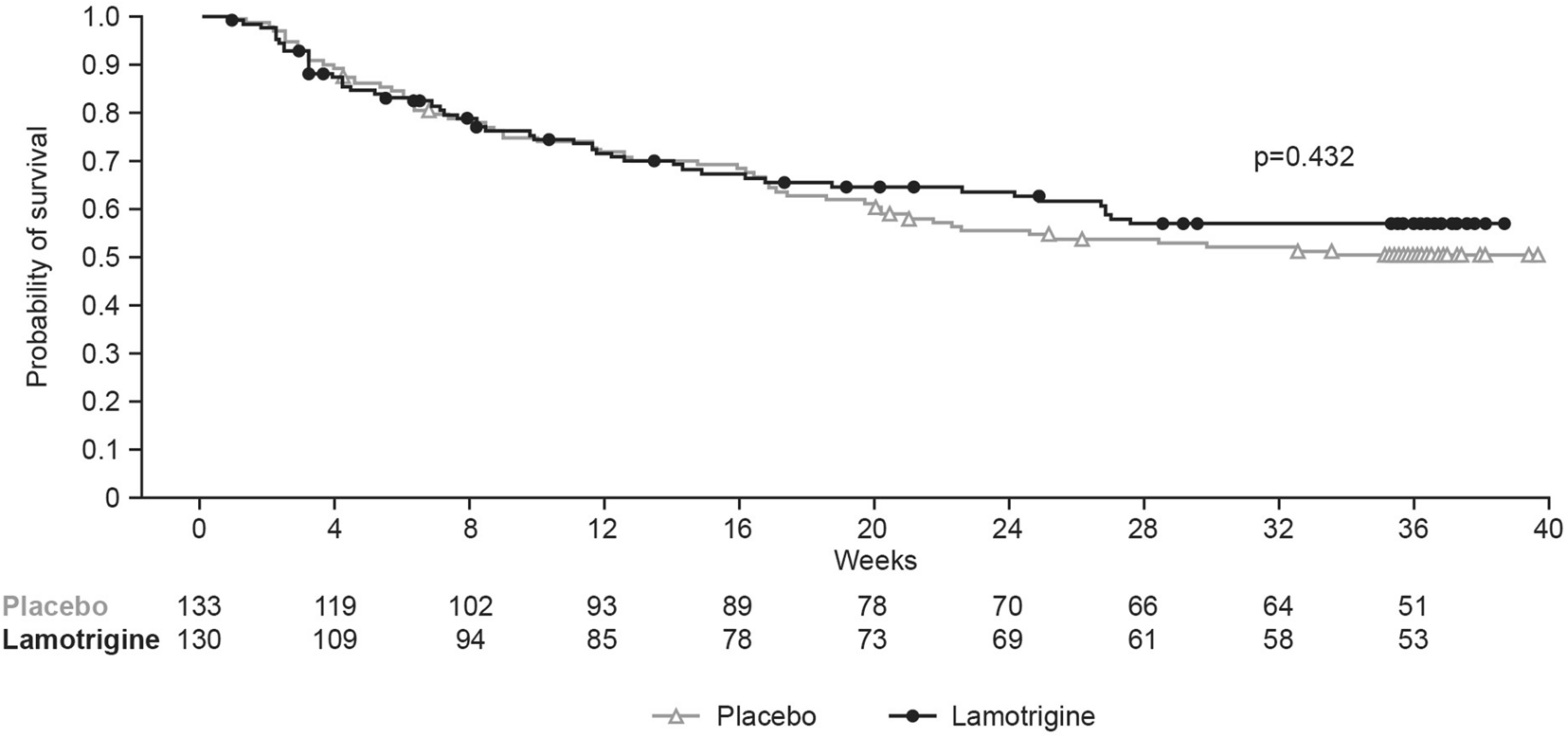
Kaplan–Meier survival estimates depicting time to intervention for mood episode (TIME). The p value was calculated using the log-rank test and is for the PBO versus LTG comparison. Marks represent censored events. Estimates are for the RD FAP. FAP, full analysis population; LTG, lamotrigine; PBO, placebo; RD, randomised double-blind; TIME, time to intervention for mood episode

Of the 264 patients who entered the RD phase, 112 patients (lamotrigine, n = 50 [38.5%]; placebo, n = 62 [46.6%]) met the primary endpoint, receiving intervention for the treatment of a relapse and/or recurrence of a mood episode. The difference in TIME between the lamotrigine and placebo arms was not statistically significant (adjusted HR [95% CI] 0.93 [0.64, 1.35]; Cox model p = 0.701). The difference was also not statistically significant when not adjusting for covariates (log-rank test p = 0.432) (Fig. 2). The median survival times could not be estimated because < 50% of patients reached the primary endpoint.

### Secondary efficacy endpoints

**Fig. 3:**
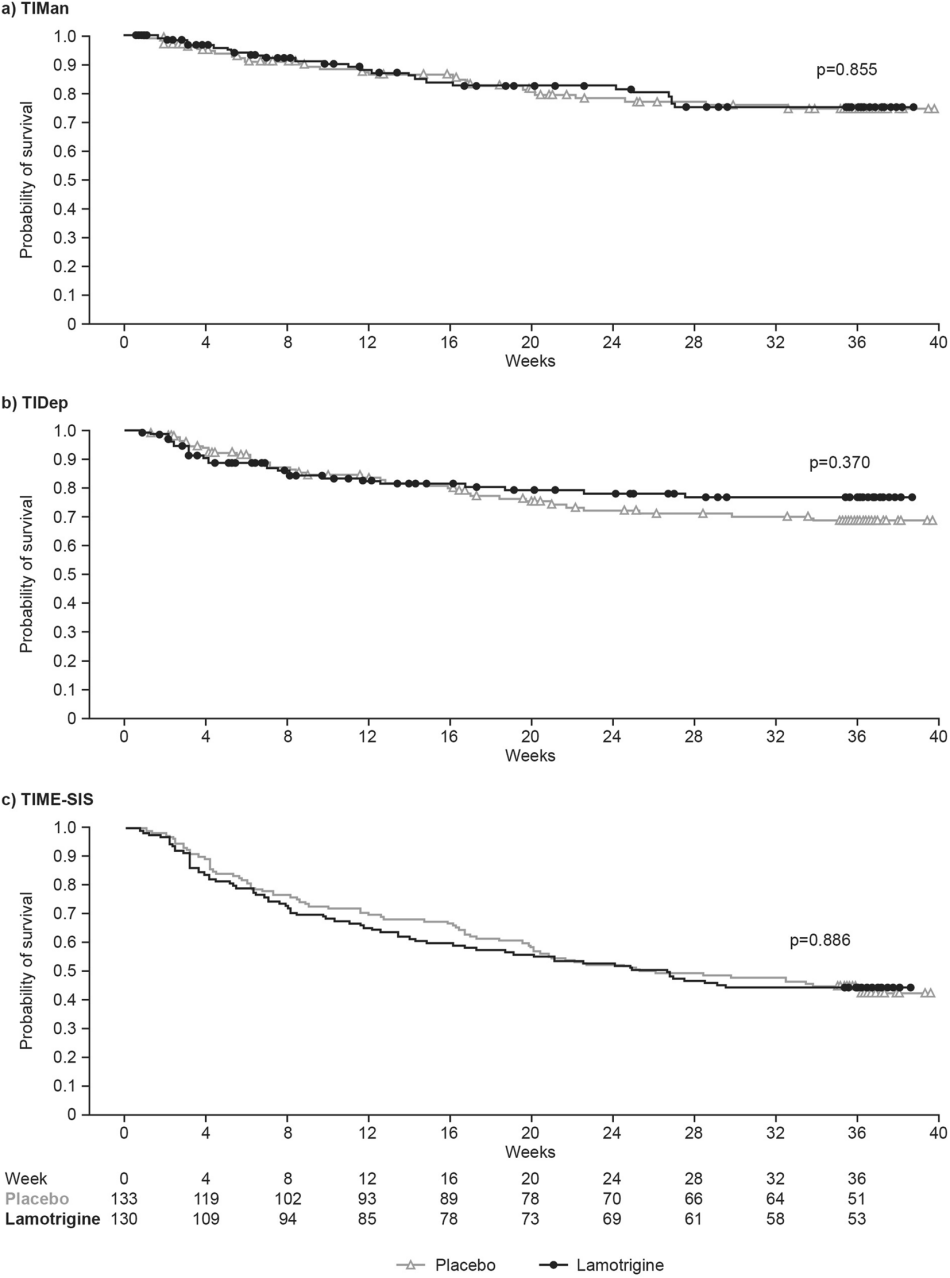
Kaplan–Meier survival estimates depicting **a** TIMan, **b** TIDep and **c** TIME-SIS. The p values were calculated using the log-rank test and is for the PBO versus LTG comparison. Marks represent censored events. Estimates are for the RD FAP. FAP, full analysis population; LTG, lamotrigine; PBO, placebo; RD, randomised double-blind; TIMan, time to intervention for manic, hypomanic or mixed episode; TIDep, time to intervention for depressive episode; TIME-SIS, overall survival in study

The Kaplan–Meier survival estimates for TIMan, TIDep and TIME-SIS for the RD phase showed no significant differences between treatment groups (p ≥ 0.370) (Fig. 3). Twenty-four (18.5%) patients from the lamotrigine group and 27 (20.3%) patients from the placebo group met the endpoint for a manic episode (adjusted HR [95% CI], 1.03 [0.59, 1.80]); 26 (20.0%) patients from the lamotrigine group and 35 (26.3%) patients from the placebo group met the endpoint for a depressive episode (adjusted HR [95% CI], 0.85 [0.51, 1.42]). Median survival times could not be estimated for TIMan and TIDep because < 50% of patients met the endpoints. For TIME-SIS in the RD FAP, a total of 72 patients (55.4%) in the lamotrigine group and 75 patients (56.4%) in the placebo group reached the event (HR [95% CI], 1.07 [0.77, 1.49]); the median survival time was 183 days for the placebo group and 188 days for the lamotrigine group.

The change from baseline in other secondary efficacy parameters (CGI-S, CGI-I, HAMD, YMRS, GAS and body weight) and quality of life (SF-36) parameters are presented in Table 2 and weekly changes from baseline in HAMD and YMRS are presented in Additional file 1: Tables S1 and S2. The difference in the change from baseline to Week 36 between lamotrigine and placebo groups was not significant (p > 0.05) for other secondary efficacy parameters, except for body weight where a larger decrease was observed amongst patients treated with lamotrigine (− 0.84 [95% CI − 1.66, − 0.01]; p = 0.047) (Table 2). Similarly, the differences in the changes from baseline to Week 36 in measures of quality of life between lamotrigine and placebo groups were not significant (p > 0.05), except for emotional role functioning (treatment difference, 0.3 [95% CI 0.0, 0.6]; p = 0.042).

**Table 2:** Change from baseline in secondary efficacy and quality of life parameters

Parameter	Treatment	N	Change from baseline, LS mean (SE)	Treatment difference (95% CI)	p value
Secondary efficacy parameters					
CGI-S	Lamotrigine	130	0.5 (0.15)	− 0.2 (− 0.5, 0.1)	0.245
	Placebo	133	0.7 (0.15)		
CGI-I	Lamotrigine	130	2.7 (0.21)	0.0 (− 0.4, 0.5)	0.833
	Placebo	133	2.6 (0.20)		
HAMD	Lamotrigine	130	1.8 (0.84)	− 1.2 (− 3.0, 0.5)	0.155
	Placebo	133	3.0 (0.83)		
YMRS	Lamotrigine	130	2.8 (0.88)	0.4 (− 1.4, 2.2)	0.661
	Placebo	133	2.4 (0.87)		
GAS	Lamotrigine	130	− 4.9 (1.77)	− 0.1 (− 3.7, 3.5)	0.945
	Placebo	133	− 4.7 (1.75)		
Body weight	Lamotrigine	128	− 1.56 (0.407)	− 0.84 (− 1.66, − 0.01)	0.047
	Placebo	130	− 0.72 (0.399)		
Quality of life parameters					
Physical functioning	Lamotrigine	127	− 0.0 (0.09)	0.0 (− 0.2, 0.2)	0.868
	Placebo	125	− 0.1 (0.09)		
Physical role functioning	Lamotrigine	127	− 0.2 (0.12)	0.1 (− 0.2, 0.3)	0.566
	Placebo	125	− 0.3 (0.12)		
Emotional role functioning	Lamotrigine	127	− 0.2 (0.15)	0.3 (0.0, 0.6)	0.042
	Placebo	125	− 0.5 (0.15)		
Social functioning	Lamotrigine	127	− 0.1 (0.13)	0.2 (− 0.1, 0.5)	0.180
	Placebo	125	− 0.3 (0.13)		
Bodily pain	Lamotrigine	127	− 0.2 (0.12)	0.1 (− 0.2, 0.3)	0.585
	Placebo	125	− 0.3 (0.12)		
Mental health	Lamotrigine	127	− 0.3 (0.15)	0.2 (− 0.1, 0.5)	0.164
	Placebo	125	− 0.5 (0.15)		
Vitality	Lamotrigine	127	− 0.1 (0.13)	0.2 (− 0.1, 0.5)	0.139
	Placebo	125	− 0.3 (0.13)		
General health perception	Lamotrigine	127	0.0 (0.11)	0.1 (− 0.1, 0.3)	0.355
	Placebo	125	− 0.1 (0.12)		

Statistical analysis performed using ANCOVA with covariates of site, CGI-S baseline score and treatment

ANCOVA Analysis of covariance; CI confidence interval; CGI-I Clinical Global Impressions of Improvement; CGI-S Clinical Global Impressions of Severity; GAS Global Assessment Scale; HAMD Hamilton Depression Rating Scale; SE standard error; YMRS Young Mania Rating Scale

Analysis population: RD full analysis population

### Safety endpoints

No deaths were reported in either the OL or RD phases of the study. Overall, 177 (42.1%) patients during the OL phase and 84 (31.8%) patients during the RD phase experienced one or more TEAEs (RD phase: lamotrigine, n = 40; placebo, n = 44) (Table 3). During the RD phase, the overall incidence of any TEAEs and treatment-related TEAEs were similar in the two treatment groups. The most common TEAEs during the OL phase were rash (4.8%), headache (4.3%) and dizziness (4.0%). The most common TEAEs reported in either group during the RD phase were nasopharyngitis (2.7%), headache, upper respiratory tract infection and urinary tract infection (2.3% each). The majority of the TEAEs reported during the RD phase in the lamotrigine (22.1%) and placebo (26.3%) groups were mild in intensity.

**Table 3:** Overall adverse events

Characteristic	Open-label phase	Double-blind phase	
	Lamotrigine N = 420	Lamotrigine N = 131	Placebo N = 133
Any AE	177 (42.1)	41 (31.3)	47 (35.3)
TEAEs related to study treatment	111 (26.4)	19 (14.5)	20 (15.0)
AEs leading to discontinuation of study treatment	40 (9.5)	5 (3.8)	5 (3.8)
Any TEAE adverse events^a^	177 (42.1)	40 (30.5)	44 (33.1)
Rash	20 (4.8)	2 (1.5)	2 (1.5)
Headache	18 (4.3)	4 (3.1)	2 (1.5)
Dizziness	17 (4.0)	2 (1.5)	1 (0.8)
Constipation	15 (3.6)	-	1 (0.8)
Fatigue	15 (3.6)	-	2 (1.5)
Nasopharyngitis	14 (3.3)	3 (2.3)	4 (3.0)
Somnolence	10 (2.4)	1 (0.8)	1 (0.8)
Hepatic function abnormal	9 (2.1)	-	1 (0.8)
Upper respiratory tract infection	9 (2.1)	2 (1.5)	4 (3.0)
Urinary tract infection	3 (0.7)	3 (2.3)	3 (2.3)
Nausea	6 (1.4)	3 (2.3)	1 (0.8)
Poor quality sleep	5 (1.2)	1 (0.8)	3 (2.3)
Weight decreased	1 (0.2)	3 (2.3)	1 (0.8)
Serious adverse events			
Any serious adverse events	12 (2.9)	2 (1.5)	3 (2.3)
Mania	5 (1.2)	–	2 (1.5)
Rash	2 (0.5)	–	–
Bipolar disorder	1 (0.2)	–	–
Ankle fracture	1 (0.2)	–	–
Face injury	1 (0.2)	–	–
Head injury	1 (0.2)	–	–
Intentional product misuse	1 (0.2)	–	–
Hepatic function abnormal	1 (0.2)	–	–
Suicide attempt	–	1 (0.8)	–
Brain stem infarction	–	1 (0.8)	–
Concussion	–	–	1 (0.8)
Contusion	–	–	1 (0.8)
Laceration	–	–	1 (0.8)

Data are presented as n (%)

TEAE treatment-emergent adverse event

Analysis population: Safety population

^a^Data for adverse events are presented for events ≥ 2% in any group, any phase

Serious AEs (SAEs) were reported for 12 patients (2.9%) in the OL phase, the most common of which was mania (n = 5; 1.2%). Five patients reported SAEs during the RD phase (placebo, n = 3 [2.3%]; lamotrigine, n = 2 [1.5%]). The SAEs in the placebo group were mania (n = 2 [1.5%]), concussion (n = 1 [0.8%]), contusion (n = 1 [0.8%]) and laceration (n = 1 [0.8%]). For the lamotrigine group, the SAEs were suicide attempt (n = 1 [0.8%]) and brain stem infarction (n = 1 [0.8%]).

A total of 40 (9.5%) patients in the OL phase and 10 (3.8%) patients in the RD phase had TEAEs leading to withdrawal from the study. The most common TEAE leading to withdrawal was rash during the OL phase (n = 17; 4.0%). During the RD phase, rash (placebo, n = 1 [0.8%]; lamotrigine, n = 1 [0.8%]) and mania (placebo, n = 2 [1.5%]) were the most common TEAE leading to withdrawal.

No clinically significant changes were observed in vital signs, ECG values, haematology, clinical chemistry or urinalysis during both the OL and RD phases. The C-SSRS assessment showed that a total of 31 (7.7%) patients reported suicidal events (ideation or behaviour) during the OL phase and 18 (7.1%) patients during the RD phase. The proportion of patients in C-SSRS Suicide Categories during the RD phase was marginally higher in the placebo group (n = 12, 9.2%) compared with that in the lamotrigine group (n = 6, 4.8%).

### Post hoc analyses

Post hoc analyses were conducted to investigate the primary efficacy endpoint (TIME) in a subgroup of patients with moderate/severe mood symptoms at baseline. The subgroup allocation by baseline mood symptom severity is shown in Additional file 1: Table S3. A total of 150 (57.0%) and 156 (59.3%) patients in the RD phase met the CGI-S and HAMD/YMRS threshold of moderate/severe baseline mood symptoms, respectively. The post hoc analyses using CGI-S or HAMD/YMRS scores both showed a greater efficacy response with lamotrigine versus placebo in patients who had more severe bipolar disorder at screening/baseline with respect to TIME.

**Fig. 4:**
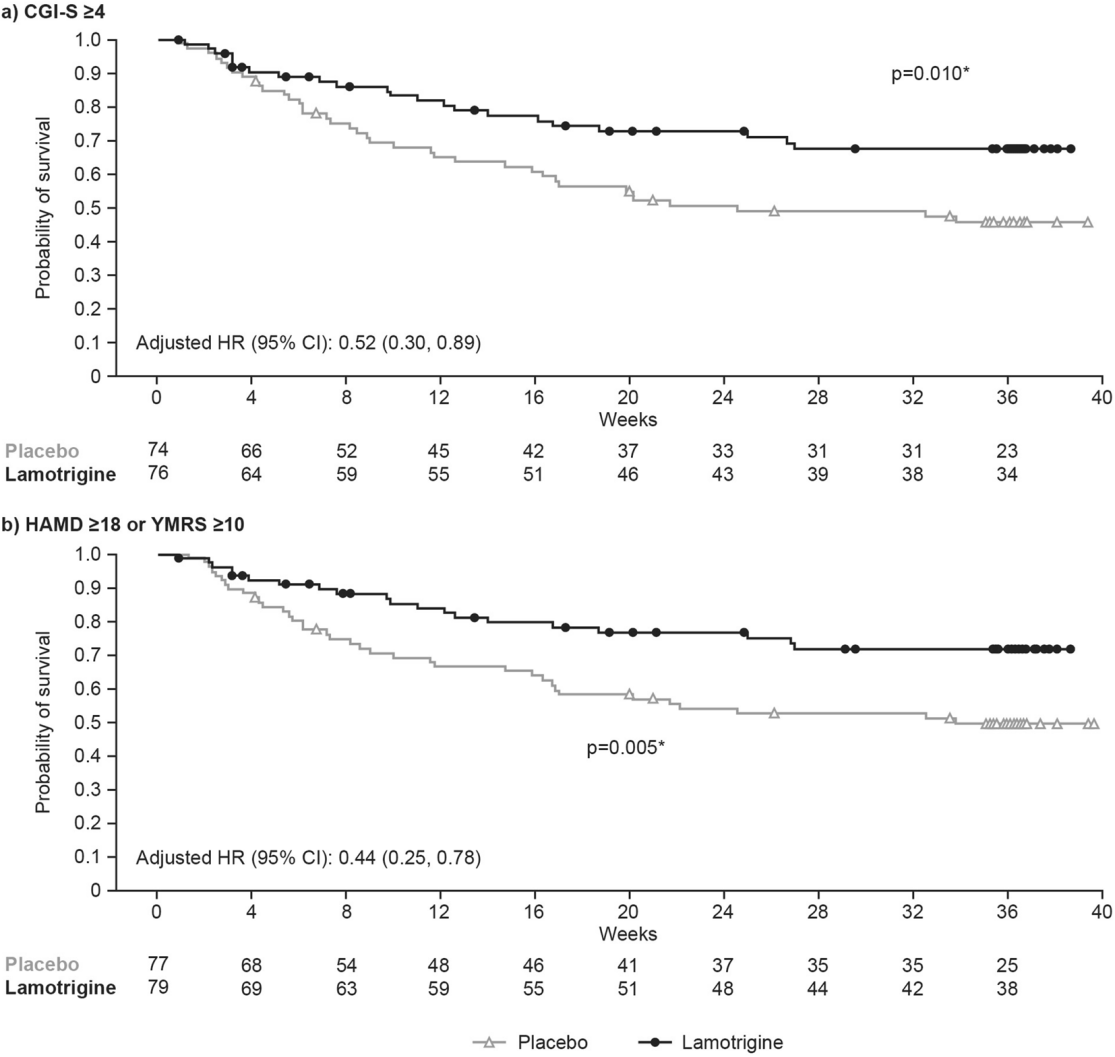
Kaplan–Meier survival estimates for TIME in patients with moderate/severe baseline mood symptoms. *The p values were calculated using the log-rank test and is for the PBO versus LTG comparison. Marks represent censored events. Estimates are for the RD FAP (excluding patients without baseline CGI-S or HAMD/YMRS scores). CGI-S, Clinical Global Impressions of Severity; FAP, full analysis population; HAMD, Hamilton Depression Rating Scale; LTG, Lamotrigine; PBO, Placebo; RD, randomised double-blind; TIME, time to intervention for a mood episode; YMRS, Young Mania Rating Scale

Of the 150 patients with baseline CGI-S ≥ 4, 60 (lamotrigine, n = 22 [28.9%]; placebo n = 38 [51.4%]) required intervention for relapse and/or recurrence of a mood episode. The difference between the lamotrigine and placebo groups using HR based on TIME was statistically significant (HR [95% CI] 0.52 [0.30, 0.89]; p = 0.018). For the 156 patients with baseline HAMD ≥ 18 or YMRS ≥ 10, 57 (lamotrigine n = 20 [25.3%]; placebo n = 37 [48.1%]) required intervention for relapse and/or recurrence of a mood episode. The treatment difference using HR based on TIME was also statistically significant (HR [95% CI] 0.44 [0.25, 0.78]; p = 0.005). Kaplan–Meier survival estimates for TIME in patients with moderate/severe baseline mood symptoms, defined using CGI-S and HAMD/YMRS scores, showed significant differences between the treatment groups (p ≤ 0.01) (Fig. 4).

Post hoc subgroup analyses by type of most recent (depressive or manic/hypomanic) or initial (depressive or mixed/manic) mood episode showed that these factors did not have a significant effect on TIME: recent depressive episode HR (95% CI) 0.96 (0.59, 1.56), p = 0.864; recent manic/hypomanic episode HR (95% CI) 0.92 (0.47, 1.82), p = 0.821; initial depressive episode HR (95% CI) 0.85 (0.49, 1.45), p = 0.546; initial mixed/manic episode HR (95% CI) 0.97 (0.47, 1.98), p = 0.932.

## Discussion

This randomised double-blind study was conducted to explore the efficacy and safety of lamotrigine for the prevention of relapse and/or recurrence of manic, hypomanic, depressive or mixed episodes in Chinese patients with bipolar I disorder. The current study was based on two earlier pivotal studies (Calabrese et al. 2003; Bowden et al. 2003), which led to the approval of lamotrigine in the global markets including the US and EU (at a target dose of 200 mg/day) (FDA 2020; Electronic Medicines Compendium 2020).

The number of patients who met the primary endpoint (relapse and/or recurrence of a mood episode requiring intervention) was slightly lower in the lamotrigine group than in the placebo group; however, this difference was not significant. Similarly, all secondary efficacy outcome measures except for body weight (TIMan, TIDep, TIME-SIS, CGI-S, CGI-I, HAMD, YMRS and GAS) did not show significant differences between the lamotrigine and placebo groups. Body weight showed a significantly greater decrease among the lamotrigine group. These findings contrast with those from the pivotal studies in international populations in which lamotrigine was superior to placebo in prolonging TIME and TIDep but not TIMan (Calabrese et al. 2003; Bowden et al. 2003). The differences in the changes from baseline in HAMD score between the lamotrigine and placebo groups in the pivotal studies were also significant, and the median survival time was significantly longer with lamotrigine versus placebo (85 vs 58 days [p = 0.03] and 200 vs 93 days [p = 0.003]) (Calabrese et al. 2003; Bowden et al. 2003). The possible reasons for the differences between these results from the pivotal studies and the current findings merit consideration.

The lack of significant efficacy in the current study could be explained by patients having a milder condition than those in previous studies, despite mostly similar inclusion and exclusion criteria (Bowden et al. 2003; Calabrese et al. 1999; Calabrese et al. 2000). Disease characteristics at baseline can have a large impact on the treatment outcome of patients with bipolar I disorder, for example, patients with a history of many mood episodes are known to respond more slowly to treatment and have a greater risk of relapse/recurrence of episodes than patients with fewer historical mood episodes (Perlis et al. 2006; Peters et al. 2014; Viguera et al. 2000). Patients who present with severe mood symptoms at baseline generally respond slowly to treatment and are likely to experience residual mood symptoms after initial treatment, the presence of which is a major risk factor for relapses/recurrences during maintenance treatment (Perlis et al. 2006; Bauer et al. 2010; Cretu et al. 2016; Judd et al. 2008, 2016; Radua et al. 2017). The patients in this Chinese study had a shorter history of bipolar I disorder and milder mood symptoms at baseline, as well as a lower CGI-S score at screening (3.6 vs 4.3 and 4.4], than in the pivotal studies (Calabrese et al. 2003; Bowden et al. 2003). Patients also had fewer depression and mania/mixed episodes in the preceding 3 years and a lower incidence of suicide attempts than in the pivotal studies (Calabrese et al. 2003; Bowden et al. 2003). Therefore, the relapse/recurrence rate of mood episodes for the placebo group during the RD phase could be anticipated to be lower than in the pivotal studies based on the severity of symptoms at baseline. Indeed, in the pivotal studies, median time to relapse on placebo was approximately 13 weeks (Calabrese et al. 2003; Bowden et al. 2003), whereas in this study only 47% of patients on placebo had relapsed by 36 weeks.

Post hoc analyses explored the potential impact of baseline symptom severity on the absence of a significant effect of lamotrigine by evaluating the primary outcome (TIME) in a subgroup of patients with moderate/severe disease at baseline defined by CGI-S or HAMD/YMRS thresholds (CGI-S score ≥ 4 or HAMD ≥ 18/YMRS ≥ 10). These post hoc data showed a statistically significant difference in favour of lamotrigine versus placebo in patients with moderate/severe mood symptoms. These results are more consistent with the observations from previously mentioned international studies (Calabrese et al. 2003; Bowden et al. 2003), but should be interpreted with caution, as the treatment difference constituted both a higher relapse rate in the placebo arm and a greater preventive effect with lamotrigine, which may suggest an increased sensitivity for detecting more severe relapses. In this context, investigators may potentially be better able to identify a severe relapse, or greater symptom severity during the index episode may serve as a clearer benchmark in an individual patient. Further post hoc analyses revealed that neither type of index episode (depressive or manic/hypomanic) nor type of initial episode (depressive or mixed/manic) affected the observed absence of a significant effect of lamotrigine on TIME.

Additional factors may also have contributed to the lack of a significant effect of lamotrigine in the main study population. The dropout rate was higher than anticipated (56% compared with the assumed 20%), further reducing the number of patients available to experience an event. Consequently, superiority of lamotrigine over placebo in preventing the relapse/recurrence of mood episodes could not be demonstrated. The duration of the RD phase within the present study was also comparatively shorter (36 weeks) compared with the previous studies (approximately 78 weeks) (Calabrese et al. 2003; Bowden et al. 2003); a longer duration of study may have resulted in a higher number of mood episodes allowing estimation of time to an event and potential demonstration of a significant effect. In a previous phase I study in healthy Chinese volunteers, lamotrigine demonstrated a comparable pharmacokinetic profile to that in the international population (FDA 2020; Li et al. 2018). Therefore, differences in genetics and ethnicity are not thought to play a major role in the differences in results between this study in Chinese patients and the studies conducted in international populations.

Overall, lamotrigine 200 mg/day showed a comparable safety and tolerability profile to placebo and led to a lower number of AEs compared to the prior studies outside China (Calabrese et al. 2003, 2008; Bowden et al. 2003; Calabrese et al. 2000). The number of patients who experienced serious TEAEs and AEs leading to withdrawal from the study were similar in both placebo and lamotrigine groups. No new or unexpected safety signals emerged based on the AEs, laboratory analyses, and monitoring of vital signs and ECGs.

This study has a number of limitations. In contrast to the pivotal studies of lamotrigine, patients were not required to have a specified type of index episode, limiting the precision with which the results can be generalised. The study may have benefitted from longer lead-in requiring patients to be stable for longer than 4 weeks. Diagnosis of bipolar disorder in this study was made without using standardised measures (e.g. Structured Clinical Interview for DSM Disorders). There was no active comparator. The use of concomitant medications during the treatment phase, including lamotrigine and valproate sodium in the RD phase, may have impacted the results.

## Conclusion

This study demonstrated that treatment with lamotrigine 200 mg/day over 36 weeks was not superior to placebo in preventing relapse and/or recurrence of mood episodes in the adult Chinese patients with bipolar I disorder who were recruited. However, post hoc analyses suggested a benefit of lamotrigine over placebo in patients with moderate/severe mood symptoms at baseline, a finding consistent with the results from previous pivotal studies. Differences in symptom severity of the bipolar patients recruited, a high-dropout rate, and the comparatively short duration of the RD phase may have contributed to the discrepancy between the negative primary findings of the present study and the positive findings of the pivotal studies on lamotrigine. Lamotrigine was well-tolerated with no new safety signals in Chinese patients with bipolar I disorder.

## Supplementary Information

**Additional file 1: Table S1.** Change from baseline in HAMD score in double-blind phase. **Table S2.** Change from baseline in YMRS score in double-blind phase. **Table S3.** Subgroup allocation by baseline mood symptom severity in open-label and randomised phases. **Figure S1.** Study design

## Data Availability

Anonymised individual participant data and study documents can be requested for further research from www.clinicalstudydatarequest.com.

## Abbreviations

AE: Adverse event
CGI-I: Clinical Global Impressions of Improvement
CGI-S: Clinical Global Impression of Severity
CI: Confidence interval
C-SSRS: Columbia Suicide Severity Rating Scale
DSM-IV: Diagnostic and Statistical Manual of Mental Disorders-IV
ECG: Electrocardiogram
EU: European Union
FAP: Full analysis population
GAS: Global Assessment Scale
GSK: GlaxoSmithKline
HAMD-17: Hamilton Depression Scale 17 Items
HR: Hazard ratio
Kg: Kilogram
LTG: Lamotrigine
OL: Open-label
PBO: Placebo
QTc: Corrected QT interval
RD: Randomised double-blind
SAE: Serious adverse event
SD: Standard deviation
TIDep: Time to intervention for depressive episode
TIMan: Time to intervention for manic, hypomanic or mixed episode
TIME: Time to intervention for a relapse or recurrence of a mood episode
TIME-SIS: Overall survival in the study
US: United States
YMRS: Young Mania Rating Scale
