# Supplementary material for: A randomised, double-blind, placebo-controlled study to evaluate the safety and efficacy of lamotrigine in the maintenance treatment of Chinese adult patients with bipolar I disorder
Source: Int J Bipolar Disord. 2022 Aug 1;10:20. doi: 10.1186/s40345-022-00266-4 (PMC9339436; doi:10.1186/s40345-022-00266-4)

**A randomised, double-blind, placebo-controlled study to evaluate the safety and efficacy of lamotrigine in the maintenance treatment of Chinese adult patients with bipolar I disorder**

Ling Zhang^a^, Honggeng Zhang^b^, Lu-xian Lv^c^, Qingrong Tan^d^, Xiufeng Xu^e^, Jian Hu^f^, Lu Zi^g^, James Cooper^h^, Abhay Phansalkar^i^, Gang Wang^a*^

^a^The National Clinical Research Center for Mental Disorders & Beijing Key Laboratory of Mental Disorders, Beijing Anding Hospital & the Advanced Innovation Center for Human Brain Protection, Capital Medical University, Beijing, China;

^b^Department of Psychiatry, Brains Hospital of Hunan Province, Changsha, China;

^c^Department of Psychiatry, Henan Mental Hospital, The Second Affiliated Hospital of Xinxiang Medical University, Xinxiang, PR China;

^d^Department of Psychiatry, Xijing Hospital, The Fourth Military Medical University, Xi’an, China;

^e^Department of Psychiatry, First Affiliated Hospital of Kunming Medical University, Kunming, PR China;

^f^The First Affiliated Hospital of Harbin Medical University, Nangang, Harbin, PR China;

^g^GlaxoSmithKline R&D Co., Ltd, Shanghai, China;

^h^GlaxoSmithKline R&D Ltd, Brentford, Middlesex, UK;

^i^GlaxoSmithKline India Global Services Private Ltd, Mumbai, India.

***Corresponding author:**

Dr. Gang Wang, Beijing Anding Hospital,
No. 5, Ankang Hutong, Xicheng District, Beijing 100088, China.
E-mail: gangwangdoc@ccmu.edu.cn

# Table S1: Change from baseline in HAMD score in double-blind phase

| **Week** | **Change from baseline** | | | | **Treatment difference** | |
| --- | --- | --- | --- | --- | --- | --- |
|  | **Lamotrigine** | | **Placebo** | | **Lamotrigine vs Placebo** | |
|  | **n** | **LS-mean (SE)** | **n** | **LS-mean (SE)** | **Difference**  **(95% CI)** | **p-value** |
| 1 | 129 | -0.3 (0.35) | 130 | -0.4 (0.35) | 0.0 (-0.69, 0.74) | 0.949 |
| 2 | 126 | 0.6 (0.41) | 128 | 0.6 (0.40) | 0.0 (-0.81, 0.87) | 0.947 |
| 3 | 120 | -0.3 (0.49) | 124 | 0.0 (0.49) | -0.3 (-1.26, 0.66) | 0.533 |
| 4 | 106 | -0.2 (0.46) | 118 | 0.2 (0.43) | -0.4 (-1.28, 0.55) | 0.433 |
| 6 | 104 | -0.5 (0.38) | 110 | -0.3 (0.38) | -0.2 (-0.98, 0.55) | 0.577 |
| 8 | 95 | -0.2 (0.44) | 103 | -0.3 (0.44) | 0.2 (-0.73, 1.06) | 0.724 |
| 12 | 87 | -0.3 (0.59) | 96 | 0.4 (0.58) | -0.7 (-1.91, 0.49) | 0.245 |
| 16 | 80 | -0.3 (0.50) | 91 | 0.8 (0.49) | -1.1 (-2.09, -0.03) | 0.044 |
| 20 | 73 | -0.8 (0.55) | 82 | 0.2 (0.54) | -1.1 (-2.21, 0.10) | 0.073 |
| 24 | 68 | 0.4 (0.52) | 72 | 0.2 (0.50) | 0.2 (-0.88, 1.35) | 0.682 |
| 28 | 65 | -0.3 (0.52) | 66 | -1.4 (0.51) | 1.1 (-0.06, 2.19) | 0.063 |
| 32 | 56 | -1.2 (0.42) | 63 | -0.7 (0.41) | -0.5 (-1.39, 0.35) | 0.240 |
| 36 | 57 | -1.2 (0.35) | 57 | -0.8 (0.35) | -0.5 (-1.28, 0.32) | 0.235 |

Analysis performed using ANCOVA with covariates of site, CGI-S baseline score, treatment and HAMD total baseline score.

ANCOVA, Analysis of covariance; CI, confidence interval; CGI-S, Clinical Global Impressions of Severity; HAMD, Hamilton Depression Scale; LS, least-squares; SE, standard error.

# Table S2: Change from baseline in YMRS score in double-blind phase

| **Week** | **Change from baseline** | | | | **Treatment difference** | |
| --- | --- | --- | --- | --- | --- | --- |
|  | **Lamotrigine** | | **Placebo** | | **Lamotrigine vs Placebo** | |
|  | **n** | **LS-mean (SE)** | **n** | **LS-mean (SE)** | **Difference**  **(95% CI)** | **p-value** |
| 1 | 129 | -0.4 (0.19) | 130 | -0.2 (0.19) | -0.1 (-0.53, 0.24) | 0.451 |
| 2 | 126 | 0.2 (0.21) | 128 | 0.2 (0.21) | -0.0 (-0.46, 0.41) | 0.900 |
| 3 | 120 | 0.2 (0.32) | 124 | 0.3 (0.32) | -0.1 (-0.69, 0.55) | 0.826 |
| 4 | 106 | 0.1 (0.28) | 118 | 0.4 (0.27) | -0.3 (-0.85, 0.28) | 0.328 |
| 6 | 104 | 0.0 (0.40) | 110 | 0.2 (0.40) | -0.2 (-1.01, 0.57) | 0.584 |
| 8 | 95 | -0.2 (0.31) | 103 | 0.1 (0.31) | -0.2 (-0.87, 0.39) | 0.454 |
| 12 | 87 | 0.7 (0.37) | 96 | 0.6 (0.36) | 0.2 (-0.57, 0.92) | 0.636 |
| 16 | 80 | 0.6 (0.55) | 91 | 0.2 (0.54) | 0.4 (-0.75, 1.53) | 0.500 |
| 20 | 73 | -0.3 (0.45) | 82 | 0.2 (0.43) | -0.5 (-1.44, 0.42) | 0.282 |
| 24 | 68 | 0.2 (0.24) | 72 | -0.2 (0.23) | 0.4 (-0.14, 0.88) | 0.153 |
| 28 | 65 | 1.7 (0.89) | 66 | 0.0 (0.87) | 1.7 (-0.18, 3.64) | 0.075 |
| 32 | 56 | -0.2 (0.24) | 63 | -0.2 (0.23) | -0.0 (-0.50, 0.47) | 0.941 |
| 36 | 57 | -0.2 (0.25) | 57 | -0.0 (0.25) | -0.1 (-0.69, 0.43) | 0.638 |

Analysis performed using ANCOVA with covariates of site, CGI-S baseline score, treatment and YMRS total baseline score.

ANCOVA, Analysis of covariance; CI, confidence interval; CGI-S, Clinical Global Impressions of Severity; YMRS, Young Mania Rating Scale; LS, least-squares; SE, standard error.

# Table S3: Subgroup allocation by baseline mood symptom severity in open- label and randomised phases

|  | **OL phase full analysis population** | **RD phase full analysis population** | | |
| --- | --- | --- | --- | --- |
|  | **Lamotrigine**  **(n=416)** | **Placebo**  **(n=133)** | **Lamotrigine**  **(n=130)** | **Total**  **(n=263)** |
| CGI-S≥4, n (%) | 243 (58.4) | 74 (55.6) | 76 (58.5) | 150 (57.0) |
| HAMD ≥18 or YMRS ≥10, n (%) | 257 (61.8) | 77 (57.9) | 79 (60.8) | 156 (59.3) |

CGI-S, Clinical Global Impressions of Severity; HAMD, Hamilton Depression Scale; YMRS, Young Mania Rating Scale; OL, open-label; RD, randomised double-blind.

# Figure S1: Study design

#
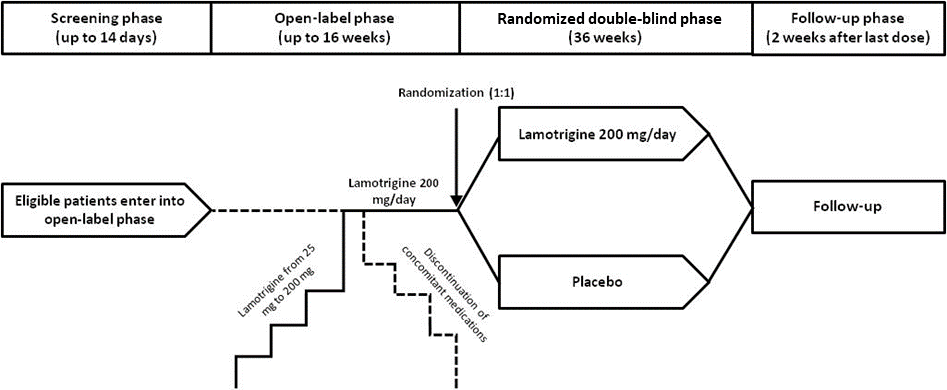

Supplement: Supplementary file 1 — Additional file 1: Table S1. Change from baseline in HAMD score in double-blind phase. Table S2. Change from baseline in YMRS score in double-blind phase. Table S3. Subgroup allocation by baseline mood symptom severity in open-label and randomised phases. Figure S1. Study design [file 40345_2022_266_MOESM1_ESM.docx]
